# Quadruplex Integrated DNA (QuID) Nanosensors for Monitoring Dopamine

**DOI:** 10.3390/s150819912

**Published:** 2015-08-13

**Authors:** Jennifer M. Morales, Christopher G. Skipwith, Heather A. Clark

**Affiliations:** Department of Pharmaceutical Sciences, Northeastern University, 206 The Fenway, 360 Huntington Avenue, Boston, MA 02115, USA; E-Mails: morales.je@husky.neu.edu (J.M.M.); c.skipwith@neu.edu (C.G.S.)

**Keywords:** dopamine, tyrosinase, enzyme, nanosensor, DNA, dendrimer

## Abstract

Dopamine is widely innervated throughout the brain and critical for many cognitive and motor functions. Imbalances or loss in dopamine transmission underlie various psychiatric disorders and degenerative diseases. Research involving cellular studies and disease states would benefit from a tool for measuring dopamine transmission. Here we show a Quadruplex Integrated DNA (QuID) nanosensor platform for selective and dynamic detection of dopamine. This nanosensor exploits DNA technology and enzyme recognition systems to optically image dopamine levels. The DNA quadruplex architecture is designed to be compatible in physically constrained environments (110 nm) with high flexibility, homogeneity, and a lower detection limit of 110 µM.

## 1. Introduction

Dopamine is a neurotransmitter that controls a wide range of cognitive functions including: motivation, behavior, reward-based learning, and motor skills [1,2]. Dopaminergic pathways are widely innervated throughout the brain and are known to modulate the behavior of other neural systems [3]. Imbalances in dopamine levels are thought to cause behavioral disorders such as schizophrenia, autism and depression. Additionally, the degradation of dopaminergic pathways plays a key role in the pathology of neurodegenerative disorders, such as Alzheimer’s, Huntington’s, and Parkinson’s disease [3,4]. Because of the great interest in the progression of these conditions that widely affect the population, there is a need for methods to continuously sense dynamic changes in dopamine. The most popular methods for sensing dopamine include microdialysis and electrochemical detection due to their unsurpassed detection limits and low cost, respectively. Of note, recently, nanopipette electrodes have been shown to detect micromolar concentrations of neurotransmitters in the synaptic cleft [5]. Capillary microdialysis coupled with liquid chromatography–mass spectrometry can analyze minute changes in cerebrospinal fluid and differentiate the types of neuropeptides present [6]. However, implantable techniques have limits on the spatial and temporal information they provide on cellular processes [7]. In comparison, fluorescent imaging techniques, such as voltage sensitive dyes, have been used in conjunction with transparent electrodes to measure changes in induced neural activity [8]. Fluorescent biosensors in particular have shown unparalleled spatial-temporal resolution of molecular processes in synapses. Optical nanosensors have a lower signal to noise ratio in comparison to small molecule fluorescent indicators [9]. Here, we combine the spatial resolution of fluorescent biosensors with the selectively of enzymes to create a novel DNA sensor platform.

DNA has become an advantageous material giving rise to sensors and devices with well-defined, specific, and robust assemblies [10]. DNA structures are utilized for self-assembly strategies and complex structures, [11] from drug delivery applications [12] to nano-mechanical devices [13]. Oligonucleotides have been demonstrated in buildable structures and various self-assembling arrangements known as DNA origami, first introduced as “staples” or short synthetic oligonucleotides to reorganize DNA sequences into simple designs [14]. Since then, DNA origami has moved from macro to nano-sized complexes, nanotubes as three dimensional arrangements [15], boxes with controllable lids, and even dolphin-shaped nanostructures with movable tails [11,16]. Recently, DNA technology has led to nanofabrication techniques that are comparable to microarray and electrode microfabrication [17]. The intrinsic nature of DNA assembly easily generates complex and uniform structures for nano and biosensing applications, such as quantum dot micro arrays [18] and nanoelectric circuits [19].

DNA-based sensors have been designed for sensing specific DNA fragments and gene sequences using short sequences of complementary DNA oligomers. For example, electrochemical sensors bind DNA to electrode surfaces for gene sequencing or alternatively use DNA coated particles for protein recognition [10,20,21]. In addition, DNA origami-based sensors have furthered sensing efficacy by combining the designability of DNA with the mechanical or functional properties of nanosensors [22]. Current DNA origami sensors have been developed for biological targets such as growth factors [23], pH [13] and lymphoma [24].

Here, we show a new method to optically detect dopamine in the low millimolar range with QuID nanosensors. These nanostructures exploit the structural motifs of DNA and biological specificity of enzymes with the size and responsiveness of nanoparticle-based sensors [25,26]. Short strands of complementary DNA strategically space sensing elements to increase the signal and intensity of the nanosensor while maintaining an approximate 100 nm diameter.

## 2. Experimental Section

### 2.1. Materials

All DNA oligomers are designed in house and commercially synthesized by AlphaDNA. The 2,2′-Azino-bis(3-ethylbenzothiazoline-6-sulfonic acid) diammonium salt (≥98% HPLC) (Azido-PEG4) was purchased from Alfa Aesar. All other chemicals and components were purchased from Sigma-Aldrich. All experiments were done at room temperature (RT), unless otherwise stated.

### 2.2. Quadruplex Integrated DNA (QuID) Design

The DNA QuID scaffold is designed to efficiently anneal into a branched structure where enzymes and fluorophores can be readily attached. Using a random sequence generator, seeding sequences were written whereby the secondary structure was determined with RNAfold nucleic acid structure prediction software using minimum energy calculations for self-assembly. Complimentary sticky ends and dendrimer subunits, designed with the use of a reverse compliment generator, were verified by RNAfold to produce a single QuID sequence. The QuID were linked together in a predictable three-tiered secondary structure by base pairing. Modifications to include guanine quadruplexes were inserted at branch points along the DNA backbone in equally spaced intervals. Enzymes are incorporated into the structure using short, complimentary, thirteen oligonucleotide sequences (5′-ATGGTGAXGCGTG-3′; X = dibenzocyclooctyl (DBCO)-dT) for site-specific tethering of enzymes along the backbone. Full QuID sequences are described and found in Supplemental Information. To create a computationally rendered DNA structure, PyMol molecular visualization system was used to combine the deposited crystal structure of *Agaricus bisporus* Mushroom Tyrosinase (PDB: 2Y9X) with the rendered DNA structure containing sequences used for construction of nanosensor scaffolding.

Attachment of tyrosinase to the DNA oligomer was done in a two-step process (Supplemental Information Figure S1). First, an Azido-PEG4 linker is attached to tyrosinase and purified; second, the DNA oligomer is attached to the opposite side of the linker by click chemistry.

### 2.3. Azido-PEG4-Tyrosinase Attachment

The Azido-PEG4 carboxyl groups were initially activated by adding 20 µM *N*,*N*,*N*′,*N*′-Tetramethyl-*O*-(*N*-succinimidyl) uronium tetrafluoroborate, 40 µM 4-(Dimethylamino)pyridine, 100 nM of Azido-PEG4 in 25 µL *N,N*-Dimethylformamide (DMF) in a centrifuge tube and vortexed at RT for one hour (Supplemental Information Figure S1a). Next, the Azido-PEG4 solution was added to 9 nM of tyrosinase in 1.9 mL phosphate buffered saline (PBS) pH 8.0 and rotated at RT for one hour. The sample was then washed 3 times using Amicron centrifuge filters (Millipore MWCO 3kD) centrifuged for 20 min at 14,000 × *g*, with PBS pH 7.4 (Supplemental Information Figure S1b). The final volume was adjusted to 60 μL with PBS pH 7.4.

### 2.4. Copper-Free Click Chemistry

Copper-free click chemistry was used to attach the azide-labeled tyrosinase to DBCO-labeled DNA (5′-ATGGTGAXGCGTG-3′) whereby the reaction was performed through mixing 25 nM of the DBCO-labeled DNA with the azide-labeled tyrosinase (Supplemental Information Figure S1c). Then the reaction mixture was rotated at RT for 6 h, washed 3 times using Amicron centrifuge filters (Millipore MWCO 3kD), centrifuged for 20 min at 14,000 × *g*, with PBS pH 7.4 and subsequently stored at 4 °C.

### 2.5. QuID Nanosensor Assembly

**Figure 1:**
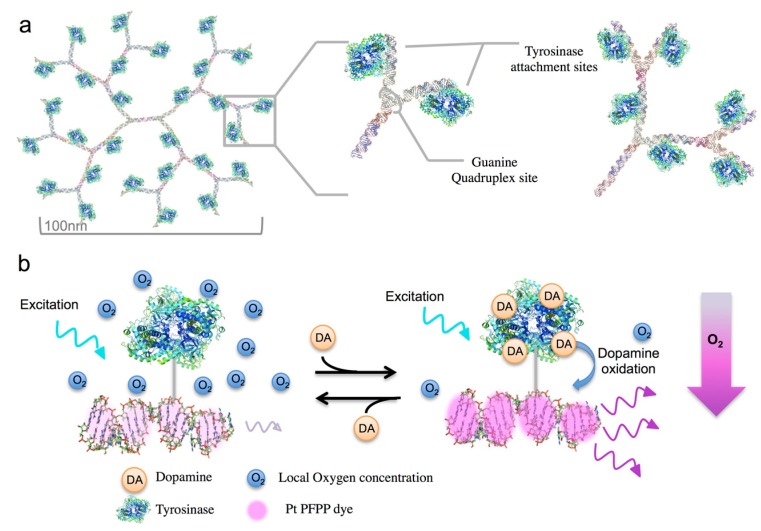
Schematic representation of (**a**) the DNA/Enzyme Nanostructure with conjugated tyrosinase tetramers and (**b**) nanosensor mechanism.

The QuID nanosensors were assembled in two main steps: DNA architecture assembly, and enzyme attachment. Assembly of DNA scaffolding was based upon the procedures of Zhou *et al.* [27], whereby using a 0.1 M phosphate buffer, pH 7.4, all DNA sequences were dissolved and heated to 90 °C for five min and cooled prior to use. Sequences used to create each subunit were mixed in equal molar amounts yielding an assembled subunit (Supplemental Information Figure S1d–e). Each QuID is comprised of three distinct subunit branches: center, first layer and second layer subunits. Combining a subunit branch with enzyme-tagged sequences assembles a full subunit (Supplemental Information Figure S1e). Then the separate subunits were added to form the tertiary nanostructure mixture in a ratio of 1:4:8 (center:layer 1:layer 2) (Figure 1a) and incubated for twenty minutes at room temperature prior to storage at 4 °C. Pt(II) meso-tetra (pentafluorophenyl) porphine (PtTPFPP) dye addition and intercalation was done at room temperature in a two-step process. First, magnesium chloride was added to the DNA solution for a final concentration of 2.5 mM. The solution was incubated at room temperature for 1 h then filtered using Amicron centrifuge filters (Millipore, MWCO 3 kDa) and 0.1 M phosphate buffer, pH 7.4. Next, PtTPFPP was added for a final concentration of 20:1 (Dye:DNA), incubated at room temperature for 1 h and filtered to remove excess dye [28]. Second, tyrosinase-tagged DNA was added in excess to the filtered DNA structure solution (12:1) and incubated at room temperature for 1 h. Last, the nanosensor solution was filtered to remove unattached enzyme-tethered DNA using Amicron centrifuge filters (Millipore, MWCO100 kDa) and 0.1 M phosphate buffer, pH 7.4. The filtered nanosensor solution was collected and stored at 4 °C.

### 2.6. Transmission Electron Microscopy (TEM) Structural Determination

Samples of complete nanosensors were prepared for TEM using the procedure described by Bock *et al.* [29]. First, the QuID nanosensors were washed with distilled water until the structure was sufficiently salt free. Then, a 300-mesh carbon film-coated copper grid (Electron Microscopy Sciences) was placed on a 10 µL, 0.6 pM drop of the QuID nanosensors for 2 min. Subsequently, the grid was rinsed with distilled water three times and placed onto a 5 µL drop of 1.5% phosphotungstic acid stain (Electron Microscopy Sciences) for an additional 3 min. To remove excess liquid the grid was dried using filter paper. A JEOL 1010 TEM at 80 kV accelerating voltage was used to collect images. The images were processed with ImageJ software suite. Measurements of six nanosensors were taken from 5 images. The TEM scale was set as 100 nm using ImageJ “set scale” option and measured manually due to the QuID’s non-spherical shape. All measurements were taken along the widest axis of each nanosensor (n = 5).

### 2.7. Continuous Variation Method (Job Plot)

A Job plot was executed to determine the ratio of dye:quadruplex using methods from Kieltyka *et al.* [30]. First, stock solutions of 5 mL of each 5 µM PtTPFPP and 5 µM quadruplex DNA (QDNA) were brought up in 10 mM potassium phosphate, 49 mM potassium chloride buffer at pH 7.2. To start, 700 µL of 5 µM QDNA was measured as a baseline in a cuvette. Next, 100 µL, 5 µM PtTPFPP was added to the cuvette and the absorbance was recorded. Successive additions and absorbance recordings were done until a plateau in absorbance, or 2 mL was reached. Then, absorbance readings were taken with additions of buffer in place of the PtTPFPP. All absorbance readings were taken in triplicate from 200 to 500 nm in a quartz cuvette. Finally, this procedure was repeated with additions QDNA into PtTPFPP. Each absorbance was corrected at 275 nm and the molar fraction of PtTPFPP was plotted against the molar fraction of QDNA. A linear fit was added to data set and the linear fit intersection was recorded as the mole fraction of QDNA:PtTPFPP.

### 2.8. QuID Size Determination

The mean particle size and size distribution of the QuID nanosensors was determined by dynamic light scattering (DLS) using a 90 Plus Particle Size Analyzer (Brookhaven Instruments Corporation). Samples were diluted with filtered distilled water. Each analysis lasted for 3 min and was performed in triplicate with an angle detection of 90° and a refractive index of 1.34. Due to the high polydispersity of the QuID (0.3 ± 0.14), the average size and size distribution were determined using the Number Distribution mean, calculated using a Non-Negative Least Squares (NNLS) algorithm with a particle refractive index of 1.34. Values reported are mean ±SD.

### 2.9. Tyrosinase Activity

A Synergy H1 Hybrid Multi-Mode plate reader (BioTek) was used to determine phosphorescence of the nanosensors in the presence of dopamine from 0.1 µM to 10 mM using an excitation frequency of 395 nm and monitoring the emission at 650 nm. Phosphorescence values were recorded every second for ten minutes. The calibration curves were generated by calculating the initial rates of the kinetic response generated for each dopamine level over the first 600 s of exposure. The change in phosphorescence response was scaled using unity-based normalization:

$$x^{\prime }=\left(x-x_{min}\right)/\left(x_{max}-x_{min}\right)$$

where *x'* is the normalized value, *x* is the sample rate, *x_max_* is the steepest rate and *x_min_* is the lowest rate. All measurements were taken with fresh solutions of neurotransmitters at pH 7.4 in PBS within one hour of preparation.

### 2.10. Reversibility

Reversibility studies were carried out with a Zeiss LSM 700 confocal laser-scanning microscope, under perfusion of alternating 1 mM dopamine and PBS solutions. Microdialysis tubing (Spectra/Por^®^ *in vivo* microdialysis tubing MWCO 13 kDa) was filled with nanosensors and fastened on a slide for imaging. The change in phosphoresce was defined by the delta function:

$$\frac{\text{Δ}F}{F\left(t\right)}=\left(F_{O}-F\left(t\right)\right)/F_{O}$$

The phosphorescence is expressed in percent, where the average phosphorescence across the first frame is the phosphorescence at a given time. Intensities were calculated from the images using Zen 2009 Microscope and Imaging software (Ziess).

### 2.11. Data Analysis

Limits of detection were determined by linear regression at each end of the calibration curve. The limits where calculated by dividing the standard error of the regression by the slope and multiplied by three. Nonlinear regression of the data to the Michaelis-Menten equation produced an *r*^2^ value of 0.99, for data points obtained within the limits of detection. For interference studies ANOVA was used to determine if the sensors were responsive to other neurotransmitters (*p* < 0.005; *n* = 3) and if the neurotransmitters interfered with the responsiveness of the sensors to dopamine (*p* < 0.005; *n* = 3). All analysis points were measured with a final nanosensor concentration of approximately 220 nM in triplicate.

## 3. Results and Discussion

The QuID nanosensor design incorporates a flexible two-dimensional DNA structure with a hierarchy of enzymes and fluorophores at every junction (Figure 1) [31]. This format strategically spaces recognition and reporter elements sites throughout the DNA structure to increase the enzyme amount surrounding each dye site. The nanosensor uses an oxygen-consuming enzyme, tyrosinase, as a recognition element coupled with an oxygen sensitive phosphorescent reporter *Pt(II) meso-tetra (pentafluorophenyl) porphine* (PtTPFPP) dye. The quadruplex DNA readily incorporates the porphyrin dye into its structure by π-π interactions between guanine-quartets in each quadruplex [32,33]. Three tyrosinase attachment sites surround each g-quadruplex dye intercalation site to maximize its interaction with PtTPFPP (Figure 1a). Tyrosinase produces localized changes in oxygen levels during dopamine oxidation [34,35]. Oxygen sensitive PtTPFPP responds to the local oxygen changes causing the sensor to “turn on” or increase its phosphorescence (Figure 1b).

**Figure 2:**
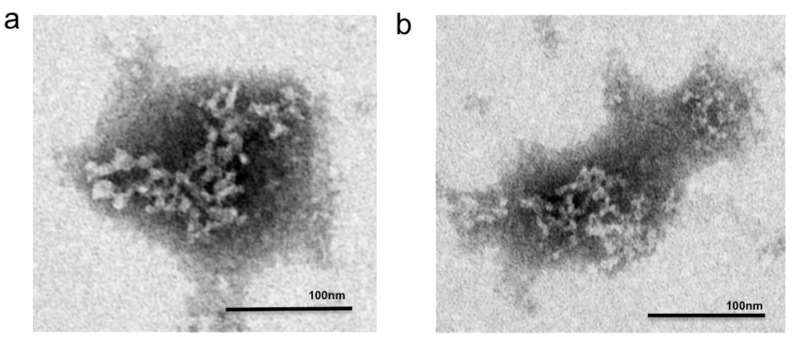
Negative stain transmission electron microscopy image of two QuID nanosensors (**a**) with visible branching and elongated shape, and (**b**) a single QuID nanosensor.

Transmission electron microscopy (TEM) provides analytical characterization of the nanosensors’ size and structure. The nanosensor assembly demonstrates the hierarchical structures with a diameter of 107 ± 30 nm, with attached 16 ± 4 nm globular extensions consistent with a multi-domain protein [36] (Figure 2). The TEM images reveal the innate flexibility of the nanosensors. Each nanosensor is approximately 110 nm long, consistent with expected diameter of 100 nm (Figure 2). QuID size was confirmed by dynamic light scattering (DLS) as 118 ± 28 nm. QuID were designed for future use as a small probe for cellular imaging in neural tissue. In order to meet the requirements necessary for this use, QuID was designed with approximate 110 nm diameter to fit within the synaptic cleft (approximate 300 nm × 300 nm × 20nm) [37]. DLS and TEM studies confirmed QuID to average 110 nm, therefore, there is potential to address small spaces in neural tissues if diffusion allows.

**Figure 3:**
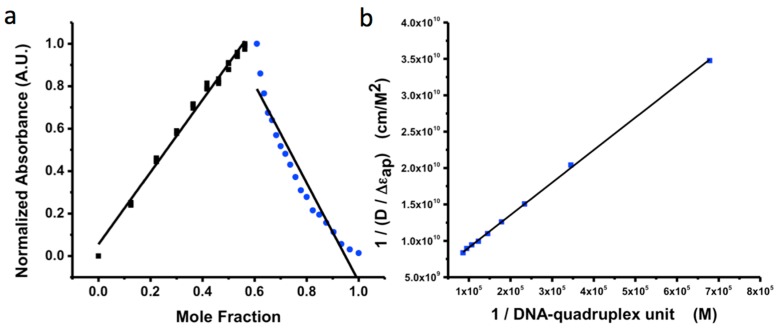
Affinity of PtTPFPP in DNA quadruplexes. (**a**) Job plot of the binding ratio (1:1) estimates 1 binding site for PtTPFPP (blue) per quadruplex unit (black); (**b**) The binding affinity of PtTPFPP and quadruplex was found to be *K* = 9.69 mM.

Guanine quadruplexes are guanine rich DNA strands that assemble predictably into stacked tetrads. Metal ions and porphyrins are known to stabilize stacked tetrads [38]. Here we use PtTPFPP to stabilize the QuID sites along the nanosensor. The continuous variation method, or Job method, quantifies the binding stoichiometry of PtTPFPP per quadruplex (Figure 3). Here, the absorbance peak of dye:quadruplex ratio shows reaction equilibrium at 0.5 M fractions. This estimates that the optimal mole ratio of binding stoichiometry is 1:1 in PBS. To increase phosphorescence response in a confined area, tyrosinase was designed to be in close proximity to the oxygen-sensitive dye. This spacing is important to maximize the response to localized oxygen depletion rather than bulk effects, but this does not entirely prevent interferences from other oxygen-consuming species.

**Figure 4:**
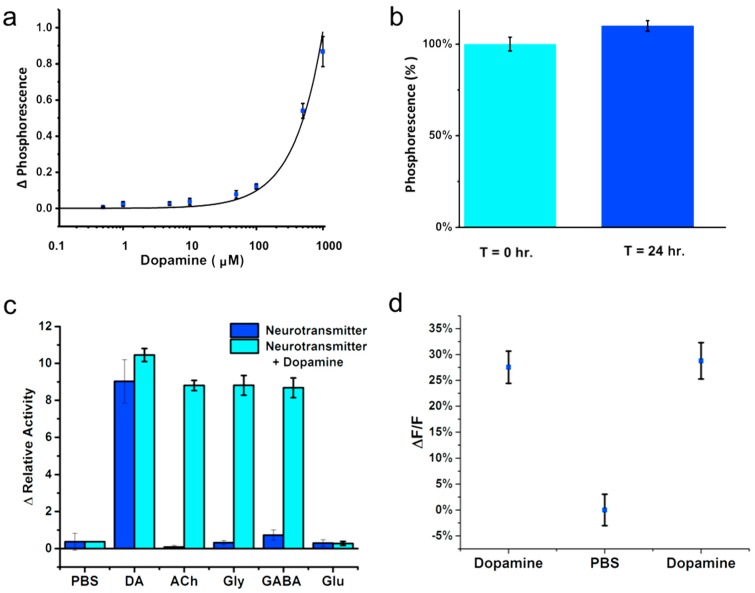
The nanosensors’ sensitivity and selectivity were measured using phosphorescence spectroscopy. (**a**) Response of dopamine sensing nanosensors from 0.01 μM^–1^·mM dopamine with a limit of detection of 110 μM (Δ Phosphorescence rate); (**b**) The nanosensors showed minimal dye leaching in solution and were stable over 24 h of consecutive phosphorescence measurements; (**c**) The selectivity of the nanosensors was examined by exposing the nanosensors to 10 mM neurotransmitter solutions of GABA, L-glutamate (Glu), glycine (Gly), and acetylcholine (ACh). Subsequent additions of 10 mM dopamine were added to confirm recovery of nanosensors activity. The neurotransmitters did not display changes in activity; except L-glutamate inhibited tyrosinase as expected; (**d**) The QuID phosphorescent response and recovery to cycles of 1 mM dopamine and PBS pH 7.4.

Sensitivity and selectivity measurements determine the accuracy of QuID nanosensors, where the change in rate of intensity per minute correlates with the amount of substrate present. Dopamine levels vary from 250 mM to 100 nM in the process of traveling from vesicles to post-synaptic neurons [39,40]. Recent estimations of basal dopamine levels required to saturate D2 receptors on the post-synaptic neuron are as high as 10 µM [41] to 100 µM [42]. The QuID are sensitive to dopamine with a 110-µM limit of detection. Notably in solution, the oxidation of dopamine results in the generation of dopamine-o-quinone, which oxidizes into dark aminochrome derivatives [43,44]. This precipitate blocks the optical signal of the sensor, which hinders us from imaging lower concentrations. Despite the limited lower limit of detection, response to dopamine over 110 µM confines QuIDs phosphorescence to areas of high dopamine levels. Thus, base levels of dopamine are not sensitive to QuID, eliminating potential false positives [45]. Additionally, the stability of the QuID was tested over 24 h to show the nanosensors are stable and do not display dye leaching from the g-quadruplexes (Figure 4b).

Nanosensor selectivity was assessed to determine if the QuID respond to other neurotransmitters or if neurotransmitters interfere with the responsiveness of the nanosensors to dopamine. First, QuID activity was recorded in the presence of 10 mM glycine, acetylcholine, GABA, and L-glutamate (Figure 4c). Second, activity was monitored for changes in fluorescence rate due to concentrations of the various neurotransmitters (10 mM), *p* < 0.005 (*n* = 3) with the addition of dopamine, final concentration of 10 mM, *p* < 0.005. We found the nanosensors are selective for dopamine and do not respond to other neurotransmitters (*p* < 0.005). Similarly, neurotransmitters show no interference on nanosensor activity; with the exception of L-glutamate (Figure 4c), which inhibits tyrosinase, leading to decreased activity. As expected, the kinetic activity of the QuID decreases due to this inhibition. To demonstrate the dynamic nature of QuID, nanosensors were immobilized inside dialysis tubing, secured to glass coverslip and imaged while perfusing solutions that alternated between 0 and 1 mM dopamine in PBS pH 7.4 (Figure 4d). As 1 mM dopamine diffuses into the dialysis tubing, it causes an increase in phosphorescence and the signal reverses when PBS (no dopamine) is subsequently perfused into the imaging chamber (Supplemental Information Figure S2). This demonstrates reversibility in the QuIDs response to dopamine, thus making it useful for dynamic imaging. Although QuID responded dynamically to dopamine, the byproduct of dopamine oxidation caused an irreversible darkening effect limiting detection of low dopamine levels. This issue was minimized in the reversibility studies where a constant perfusion cleared any residual dopaminechrome. In comparison, off-line methods of monitoring dopamine concentrations, such as microdialysis and High-Performance Liquid Chromatography, likewise remove the dopaminechrome before measurement [46]. The darkening effect will be an important consideration in cultured tissue since it is difficult to remove dopaminechrome as it is produced, and will be the target of future studies as these sensors are used for measurements. The QuID, therefore, meet all requirements in size, selectivity and reversibility making it suitable for spatially monitoring peak dopamine levels.

## 4. Conclusions/Outlook

We have shown optical QuID nanosensor selectivity and reversibility to peak synaptic dopamine levels. This nanosensor utilizes a “turn on” sensing method where it phosphoresces in the presence of dopamine. To become an effective imaging tool, QuID would benefit from enzymes for aminochrome clearance and D2 dopamine receptor antagonists for localization to synaptic regions of dopaminergic neurons. Additionally, this nanosensor can be paired with a transparent electrode-based sensor for combined spatial-temporal resolution of dopamine dynamics.

## Supplementary Files

Supplementary File 1
