# Supplementary material for: Quadruplex Integrated DNA (QuID) Nanosensors for Monitoring Dopamine
Source: Sensors (Basel). 2015 Aug 13;15(8):19912–24. doi: 10.3390/s150819912 (PMC4570402; doi:10.3390/s150819912)
Supplement: Supplementary File 1 [file sensors-15-19912-s001.pdf]

OPEN ACCESS

sensors

ISSN 1424-8220

www.mdpi.com/journal/sensors

Supplementary Information

Quadruplex Integrated DNA (QuID) Nanosensors for Monitoring Dopamine. *Sensors* 2015, *15*, 19912-19924

Jennifer M. Morales, Christopher G. Skipwith and Heather A. Clark *

Department of Pharmaceutical Sciences, Northeastern University, 206 The Fenway, 360 Huntington Avenue, Boston, MA 02115, USA; E-Mails: morales.je@husky.neu.edu (J.M.M.); c.skipwith@neu.edu (C.G.S.)

* Author to whom correspondence should be addressed; E-Mail: h.clark@neu.edu; Tel.: +1-617-373-3091.

DNA sequences used to produce the nanosensor. All sequences are shown in the 5′ to 3′ direction.

Center Sequences

GCTAGGGGTCCTTGGGGGGCGAGGAGGAGGCT

ATCATACTAATCGCACGCATCACCATAGCCTCCTCCTCGGGGGGG

GGGGGGTAAACTTTCCTAG

ATCATACTAATCGCACGCATCACCATCTAGGAAAGTTTAGGGGGG
AAGGACCCCTAGCCCGCAGCGAACAT

TTCAGTTTTTAACGGGGGGCCATGGTACCCTC

ATCATACTAATCGCACGCATCACCATGAGGGTACCATGGGGGGGG

GGGGGGGGTCTTTCTCCTG

ATCATACTAATCGCACGCATCACCATCAGGAGAAAGACCGGGGGG
GTTAAAAACTGAAATGTTCGCTGCGG

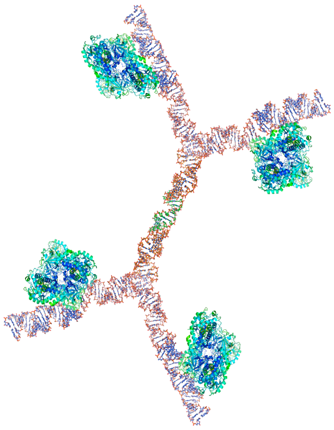

Features

Complementary sticky ends for binding central pieces

CCGCAGCGAACAT ATGTTCGCTGCGG

Common sticky end to bind Layer 1

ATCATACTAATCG

Guanine Quadruplex coupling region

GGGGGG

Enzyme tethering region and complementary DNA sequence for enzyme attachment

CACGCATCACCAT ATGGTGATGCGTG

Layer 1 Sequences

CGATTAGTATGATTGATGCTAACTTAGGGGGGTTAGACGATGCTG

TCAACTTTCCCTCCACGCATCACCATCAGCATCGTCTAAGGGGGG

GGGGGGAGGGACGTCGCGT

TCAACTTTCCCTCCACGCATCACCATACGCGACGTCCCTGGGGGGTAAGTTAGCATCA

Features

Complementary sticky ends for binding to center piece

CGATTAGTATGAT

Common sticky end to bind Layer 2

TCAACTTTCCCTC

Guanine Quadruplex coupling region

GGGGGG

Enzyme tethering region and complementary DNA sequence for enzyme attachment

CACGCATCACCAT ATGGTGATGCGTG

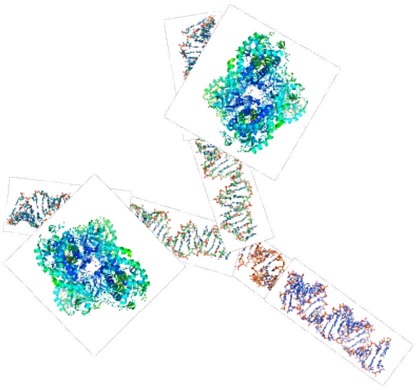

Layer 2 Sequences

GAGGGAAAGTTGATTCAGTTTTTAACGGGGGGCCTCTAATCCCCA

ATAATTCAGCGATCACGCATCACCATTGGGGATTAGAGGGGGGGG

GGGGGGCCAGAAAGAGGAC

ATAATTCAGCGATCACGCATCACCATGTCCTCTTTCTGGGGGGGGTTAAAAACTGAA

Features

Complementary sticky ends for binding to Layer 1

GAGGGAAAGTTGA

Common sticky end for additional binding

ATAATTCAGCGAT

Guanine Quadruplex coupling region

GGGGGG

Enzyme tethering region and complementary DNA sequence for enzyme attachment

CACGCATCACCAT ATGGTGATGCGTG

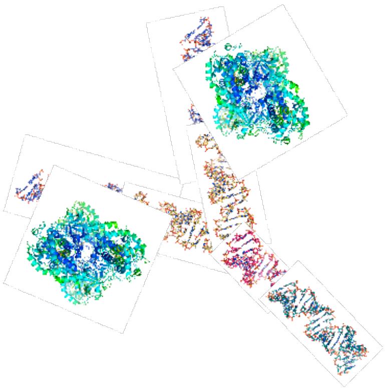

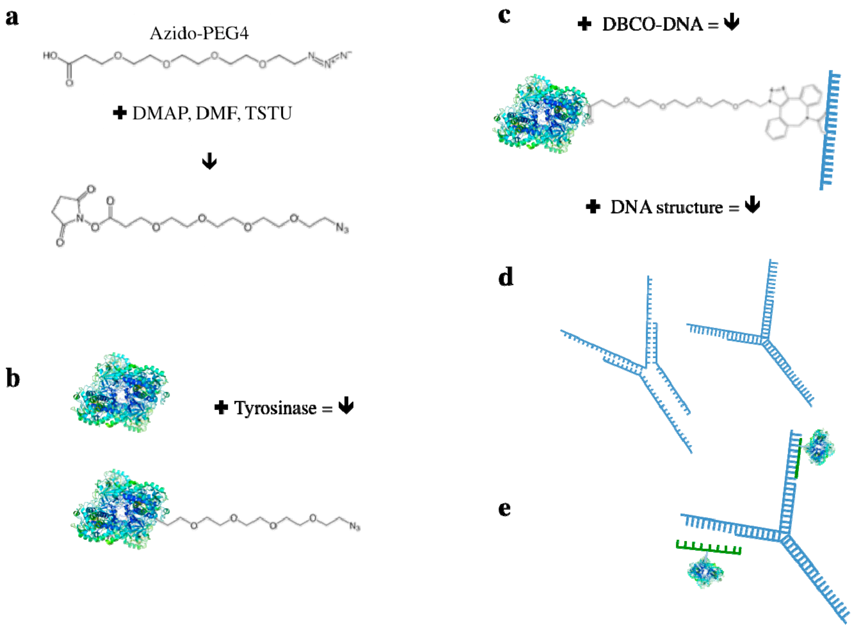

Figure S1. Schematic description of QuID assembly. The Azido-PEG4 linker is first activated (**a**) and conjugated to tyrosinase with amine reactive chemistry (**b**); Next the DNA is attached to the linker by a copper-free click chemistry reaction (**c**); Separately, the DNA structure is assembled (**d**) and mixed with the enzyme conjugated DNA (**e**).

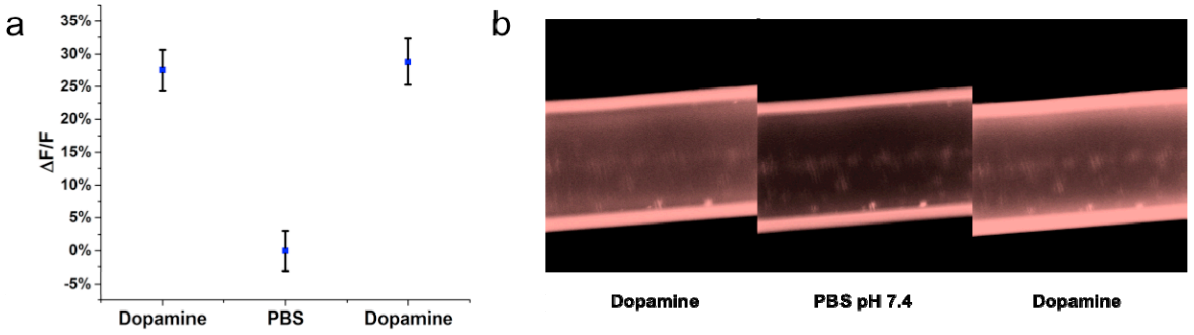

Figure S2. Reversibility of QuID phosphorescence (**a**) with corresponding images of the time points during perfusion (**b**).

© 2015 by the authors; licensee MDPI, Basel, Switzerland. This article is an open access article distributed under the terms and conditions of the Creative Commons Attribution license (http://creativecommons.org/licenses/by/4.0/).
